# An Uncommon Case of Fournier's Gangrene in a Female Patient

**DOI:** 10.7759/cureus.50906

**Published:** 2023-12-21

**Authors:** Emma N Barnes, Erika Arvelo, Michael W Fountain

**Affiliations:** 1 Urology, Lake Erie College of Osteopathic Medicine, Jacksonville, USA; 2 Urology, Lake Erie College of Osteopathic Medicine, St. Augustine, USA; 3 Urology, AdventHealth Waterman, Tavares, USA

**Keywords:** necrotizing fasciitis, fournier's gangrene in a female patient, female fournier's gangrene, fournier's gangrene (fg), fournier gangrene

## Abstract

Fournier’s gangrene is a rare form of infectious fasciitis in the genital region. It is a rapidly progressing, life-threatening infection that requires immediate diagnosis and treatment. Common risk factors for Fournier’s gangrene include diabetes mellitus, obesity, trauma, alcoholism, and cigarette smoking. The infection is more commonly seen in men than women, but we present here a case of Fournier’s gangrene in a 74-year-old woman. The incident started as a small lump in the genital region from a fall and progressed into a severe case of necrotizing fasciitis. Emergent surgical debridement and antibiotics were required, as mortality depends greatly on prompt management.

## Introduction

Fournier’s gangrene is a rare form of necrotizing fasciitis that affects the genital, perineal, or perianal regions [1]. Bacterial composition consists of a mixture of anaerobic and aerobic bacteria. A study conducted in 2009 found *Escherichia coli* and *Pseudomonas aeruginosa* as the predominantly isolated organisms in their patient population [2]. Other less prevalent isolated bacteria included *Staphylococcus aureus*, *Enterobacter cloacae*, and *Providencia rustigianii*. The primary risk factor for Fournier’s gangrene is diabetes mellitus (DM), with other risks further relating to a weakened immune system in the patient, including trauma, recent surgery, smoking, and alcoholism [3,4]. Males are more commonly affected than women. While the exact prevalence in women compared to men is unknown, a study conducted in the United States in 2016 found a male-to-female ratio of 40:1 in their pool of cases [5]. The study also found that women were generally more acutely ill than male patients at presentation to the hospital. Symptoms include pain, fever, swelling of the region, and malaise [3]. If Fournier’s gangrene is suspected, the patient must be treated immediately, as the condition can rapidly progress to sepsis and multi-organ failure [6]. Clinical examination, imaging revealing gas in the affected regions, and cultures are used for diagnosis [6]. Patients must receive broad-spectrum antibiotics and fluids as rapidly as possible, with further treatment in the form of emergent surgical exploration and staged debridement [6].

## Case presentation

Here we present a case of a 74-year-old woman who presented to the emergency department five days after falling from her shower chair. The patient remained on the floor for two hours after her fall. Upon arrival to the emergency department, she reported cold-like symptoms, weakness, bilateral knee abrasions, and a right labial lump that had developed in the time since her fall. The patient had a history of hypertension, hyperlipidemia, myocardial infarction, coronary artery disease, carotid stenosis, and cigarette smoking (1.5 packs/day). She had no history of DM. She stated that she had been unable to tolerate liquids since her fall due to nausea and vomiting. She described her labial lump as a small bump that looked like a pimple and subsequently popped, leaving persistent pain and redness in the area. On physical examination, she was tachycardic with a heart rate of 115 bpm, and her BMI was 42.43. Table 1 shows the complete blood count (CBC), which was remarkable for leukocytosis, reflecting her active infection.

**Table 1:** Complete blood count WBC, white blood cell; RBC, red blood cell; L, liter; g/dL, grams per deciliter; μm^3^, cubic micrometers; pg/cell, picograms per cell; Hb/cell, hemoglobin per cell; mm^3^, cubic millimeters; fL, femtoliters; NRBC, nucleated red blood cell count

	Laboratory Value	Reference Range
WBC count	14.97	4.5–11.0 × 10^9^/L
RBC count	4.78	4.3–5.9 × 10^12^/L
Hemoglobin	13.6	13.5-17.5 g/dL
Hematocrit	40.2	41%-53%
Mean corpuscular volume	84.1	80–100 μm^3^
Mean corpuscular hemoglobin	28.5	25–35 pg/cell
Mean corpuscular hemoglobin concentration	33.8	31%–36% Hb/cell
Red blood cell distribution width	13.2	12%–15%
Platelet count	164	150,000-400,000 mm^3^
Mean platelet volume	10.8	7.5–11.5 fL
NRBC%	0.1	0%
NRBC, absolute	0.02	0 nucleated RBC/100 WBC

The complete metabolic panel showed hyperglycemia and elevated aspartate transaminase (AST), as given in Table 2. The patient's hyperglycemia could indicate increased cortisol release due to the stress of her fall. Her AST levels possibly reflected muscle damage from her prolonged immobilization on her shower floor. She was later diagnosed with rhabdomyolysis during her hospital stay.

**Table 2:** Comprehensive metabolic panel BUN, blood urea nitrogen; A/G ratio, albumin-to-globulin ratio; eGFR, estimated glomerular filtration rate; mg/dL, milligrams per deciliter; mL/min/1.73m^2^, milliliters per minute per 1.73 meters squared; mmol/L, millimoles per liter; g/dL, grams per deciliter; U/L, units per liter

	Laboratory Value	Reference Range
Sodium	124	135–145 mmol/L
Potassium	4.1	3.5–5 mmol/L
Chloride	86	95–105 mmol/L
Carbon dioxide	18	22–32 mmol/L
Anion gap	20	4–12 mmol/L
BUN	19.0	7–25 mg/dL
Creatinine	1.20	0.7–1.5 mg/dL
BUN//creatinine ratio	15.8	6–22
Glucose	202	70–100 mg/dL
Calcium	8.7	8.5–10.5 mg/dL
Aspartate transaminase	102	10–35 U/L
Alanine transaminase	26	0–31 U/L
Alkaline phosphatase	123	25–125 U/L
Protein, total	6.4	6.5–8.1 g/dL
Albumin	2.9	3.5–5.0 g/dL
Globulin	3.5	2.0–3.5 g/dL
A/G ratio	0.8	0.8–2.0 g/dL
Bilirubin total	0.70	0.0–1.2 mg/dL
eGFR	47.6	>60 mL/min/1.73m^2^

The patient’s lactic acid level was elevated (3.80 mmol/L; reference value: 0.50-2.20 mmol/L) due to her infection and rhabdomyolysis. Her creatinine kinase levels, as shown in Table 3, were also significantly increased, further supporting her diagnosis of rhabdomyolysis.

**Table 3:** Creatinine kinase U/L, units per liter

	Laboratory Value	Reference Value
Creatinine kinase	8,368	Male: 25–90 U/L; female: 10–70 U/L

**Figure 1:**
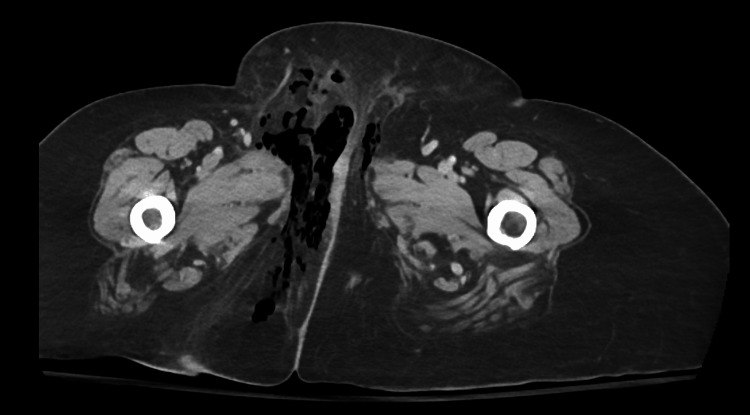
CT (axial view) with contrast of the abdomen and pelvis CT with contrast of the abdomen and pelvis showed extensive subcutaneous gas collections in the right labia, right adductors, and perineum.

**Figure 2:**
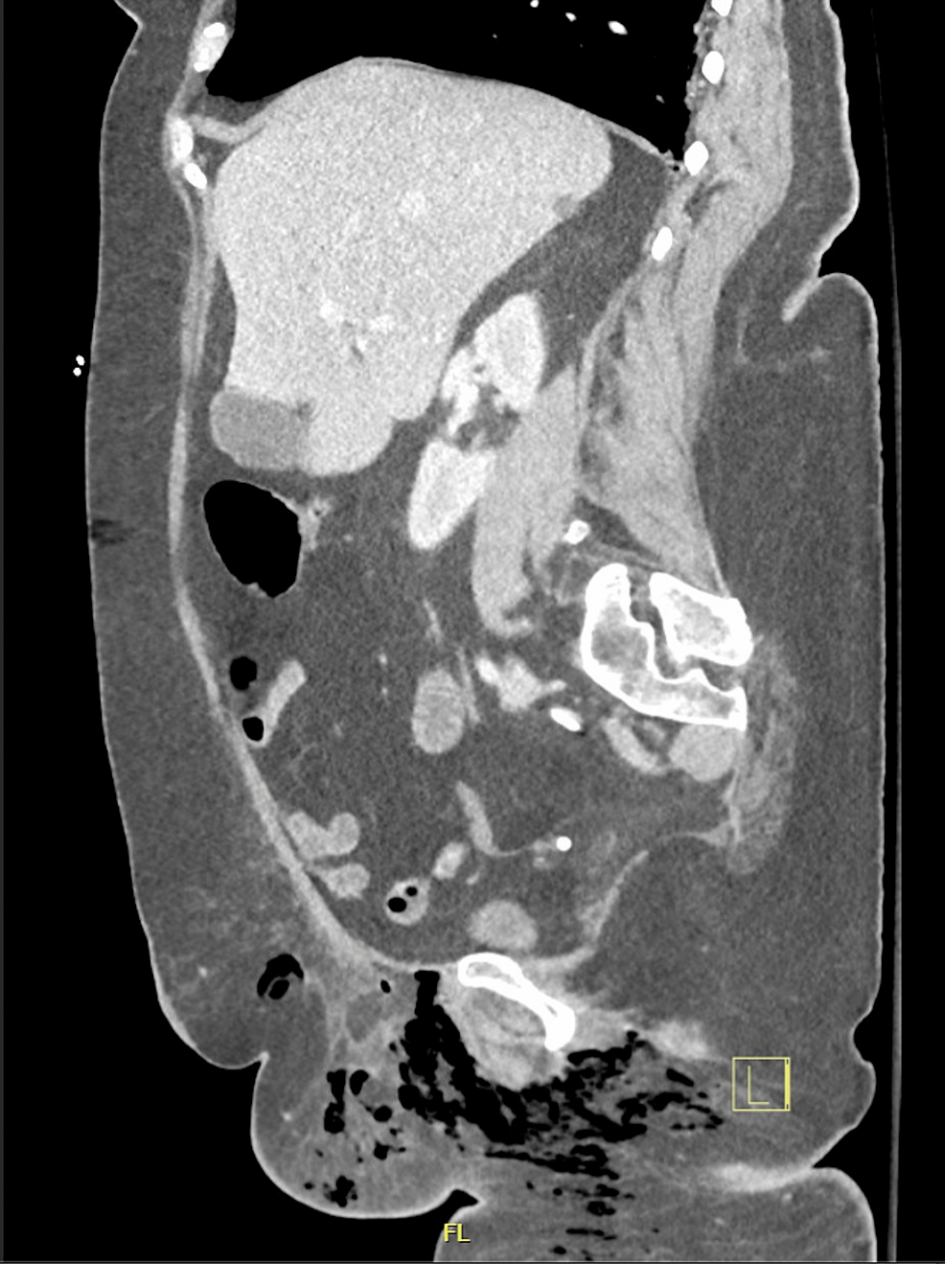
CT (sagittal view) with contrast of the abdomen and pelvis Subcutaneous gas was visible in the right labia, right adductors, and perineum.

Examination of the genital region revealed warmth, redness, and induration in the right pubic area. A CT of the abdomen and pelvis with IV contrast was performed. Results of the scan are shown in Figure 1 and Figure 2.

Due to the concern for necrotizing fasciitis, the patient was rapidly started on piperacillin-tazobactam, clindamycin, and vancomycin. A normal saline bolus was also given, and general surgery was consulted.

General surgery performed a right perineal wound debridement. The procedure revealed extensive necrotizing fasciitis involving the right labia, right perineum, and right medial thigh. Depth of infection was recorded as 18-20 cm extending from the skin to the subcutaneous tissue, muscle, and fascia. A culture was obtained, and tissue was sent to pathology. Results later revealed heavy growth of anaerobic gram-positive cocci and *Actinomyces turicensis*. The patient was admitted to the intensive care unit with planned return to operating room for staged surgical debridement.

The patient's hospital stay was complicated by atrial fibrillation with rapid ventricular response, infection with *Clostridium difficile*, and rhabdomyolysis likely due to her prolonged immobilization. Infectious disease managed the patient’s antibiotic course, with IV meropenem and oral vancomycin provided. The patient was discharged to a rehab facility with recommended weekly labs for three weeks and a follow-up with urology.

## Discussion

Fournier’s gangrene is a rapidly progressing life-threatening infection. It has been suggested that the infection has the capability to spread as fast as 2-3 cm per hour [7]. If not rapidly treated, shock, multi-organ failure, and sepsis can result [7]. The common risk factors and underlying conditions for necrotizing fasciitis also contribute to the mortality rate, with examples including DM, obesity, and trauma [8]. While DM is a largely associated risk factor for the disease, our patient had no such history. The patient's obesity served as a major risk factor for Fournier’s gangrene. Her history of smoking also made her more susceptible. Cases are more commonly found in men, exemplified by the fact that this was a novel diagnosis of female Fournier’s gangrene for the urologist consulted. Infection and necrosis in Fournier’s gangrene is the result of bacterial infection causing microthrombosis of small vessels, leading to ischemia and gangrene in the supplied tissue [9]. Early recognition of infection is crucial, as the standard mortality rate of Fournier's gangrene can increase from 40% to 88% due to delay in diagnosis and treatment [7]. While the diagnosis is primarily clinical, a thorough physical examination, labs, and imaging can greatly aid in diagnosis [7]. It is important to rule out Fournier's gangrene whenever a patient complains of pain in the genital or perineal regions, especially if the pain appears out of proportion to the physical findings, as early skin presentation can be minimal [7]. Labs commonly show leukocytosis and elevated lactic acid, while imaging such as ultrasound, X-ray, and CT can reveal subcutaneous gas accumulation [7]. Broad-spectrum antibiotics must be rapidly administered to patients [7]. Necessary coverage includes gram negatives, streptococcal, staphylococcal, pseudomonas, coliform, and clostridium species of bacteria [7]. Multiple sessions of surgical debridement are vital, with the average number of debridements needed for each patient being 3.5 [10]. Negative-pressure treatment has also been found to be helpful following staged wound debridement, as the treatment increases blood supply to the region and promotes healing through increased movement of inflammatory cells [7].

## Conclusions

Fournier’s gangrene is a severe infection primarily found in patients with a weakened immune response. Although it is more common in men, women with predisposing risk factors and presenting with redness, pain, and swelling in the urogenital region must be suspected to have Fournier’s gangrene. Early recognition is essential, with delay greatly contributing to the high mortality rate of the infection. Antibiotics and staged surgical debridement are cornerstones of treatment. A multidisciplinary approach to treatment is essential to proper management of the infection.
